# Student Perception of Teacher and Parent Involvement in Homework and Student Engagement: The Mediating Role of Motivation

**DOI:** 10.3389/fpsyg.2019.01384

**Published:** 2019-06-13

**Authors:** José C. Núñez, Bibiana Regueiro, Natalia Suárez, Isabel Piñeiro, María Luisa Rodicio, Antonio Valle

**Affiliations:** ^1^Department of Psychology, University of Oviedo, Oviedo, Spain; ^2^Department of Psychology, University of A Coruña, A Coruña, Spain; ^3^Department of Specific Didactics and Methods of Research and Diagnosis in Education, University of A Coruña, A Coruña, Spain

**Keywords:** student homework motivation and engagement, perceived parental homework involvement, perceived teacher homework involvement, secondary education, homework engagement

## Abstract

Currently, there is much debate about the value of assigning homework. Organizations such as the OECD have concluded that doing more homework is not synonymous with better performance. This study was designed to analyze the mediating role of student motivation in the relationship between the involvement of parents and teachers in homework and the engagement of students in these tasks. Seven hundred and thirty students in Compulsory Secondary Education (7th–10th grade) participated from 14 schools in the north of Spain. Three competing models were developed and tested to study motivational mediation: a non-motivational mediation model (direct effects model); a total motivational mediation model (indirect effects model); and a partial motivational mediation model (mixed effects model). The best model was adjusted according to gender and school year variables. The total mediation motivational model demonstrated the best fit (indirect effects model). The results suggest the total mediation of student motivation in the relationship between the perception of parents’ and teachers’ involvement in homework and student cognitive engagement in these tasks. Some differences, albeit slight, were observed with respect to gender and school year. The results have clear theoretical and educational implications.

## Introduction

Homework has been a very common topic in educational research in recent decades (Trautwein, 2007; Fernández-Alonso et al., 2015; Valle et al., 2015; Baş et al., 2017; Fan et al., 2017), most of which has tended to analyze its relationships and its real impact on student academic achievement. Past research has often focused more on aspects related to the amount of homework done and the time spent on it than on the quality of the homework process, its precursors, and its effects on learning.

The homework process is what students do when dealing with homework; how they approach their work and how they manage their personal resources and settings when they do homework. Research and theory suggest that students’ intentions and reasons for doing homework influence how they cope with it; in other words, the quality of their engagement (Ryan and Deci, 2000). Some students approach homework with the intention of learning and reinforcing the knowledge acquired in class, trying to resolve questions that may arise while doing homework, and relating the homework to what they have previously learned. It therefore involves an intrinsic purpose of understanding the ideas and using strategies to create meaning. In this context, intrinsic motivation has been associated with a host of positive outcomes such as persistence, performance, interest, and positive emotions (Bouffard et al., 2001; Hardre and Reeve, 2003; Coutts, 2004; Valle et al., 2016). Most studies have shown that the deeper students’ approach to learning, the better their learning outcomes. When students are involved in academic tasks mainly for the purpose of understanding, they do those tasks more profoundly and meaningfully, they use self-regulation strategies in their learning process and exhibit better well-being (Bouffard et al., 2001; Midgley, 2002; Vansteenkiste et al., 2005). Conversely, if students work on homework because they feel compelled by their teachers, and perhaps by their parents, a sense of duty or avoidance of punishment (Walker et al., 2004), it is very likely that the student will exhibit poor persistence and little significant learning (Vallerand et al., 1997).

Various theoretical approaches have been used as frameworks for research in the past: Self-Determination Theory (SDT- Deci and Ryan, 2000), Expectancy-Value Theory (EVT- Eccles, 2005), Goal Orientation Theory (Elliot, 2005) and the Student Engagement Framework (Reschly and Christenson, 2012). These theoretical frameworks, in various ways, agree in assuming that academic motivation is context-dependent. The support provided by context (mainly parents and teachers) is fundamental in explaining the type of, and changes in, motivation (Katz et al., 2010). In Connell and Wellborn’s (1991) model of self-system processes, motivation was viewed as a mediator between context and outcomes. In our study, as in recent research (e.g., Feng et al., 2019), we attempted to analyze the extent to which this model can be applied to the field of homework. The student engagement framework seems to be a good theoretical model to pursue this objective.

In this study we investigate to what extent students’ homework motivation mediates between the support of the context (i.e., parental and teacher homework involvement) and student homework engagement.

### Motivation and Student Homework Engagement

Engagement and motivation to learn are highly interrelated. Some researchers use the terms engagement and motivation interchangeably (e.g., Martin, 2007), others have proposed that the meta-construct of student engagement subsumes motivation (e.g., Fredricks et al., 2004), while others argue that they are different, but closely related constructs. As different constructs, motivation represents intention and engagement represents action (e.g., Russell et al., 2004). In our study we follow this third line of thought: motivation and student engagement are understood as related, but different constructs.

Student engagement has been significantly associated with contextual factors (Lam et al., 2012). Parent and teacher involvement are two of the main variables responsible for student motivation and homework engagement. Research has identified parent involvement in homework as one way that parents and families can influence student motivation and school engagement. Parents who provide assistance with homework play a critical role not only in fostering learning, but in scaffolding strategies for time management and problem-solving (Moè et al., 2018). Furthermore, their interest in and help with homework predicts their children’s self-perceptions of competence (Hoover-Dempsey et al., 2001; Pomerantz et al., 2006).

So, how does parent and teacher involvement in homework impact children’s engagement and achievement? To answer this primary question, we bring the proposal from Grolnick and Slowiaczek (1994) to the homework field. They suggest two models: (i) a direct effects model, and (ii) an indirect or motivational model.

Focusing on the field of parental involvement in homework, the direct effects model would suggest that parental involvement in children’s homework helps children by teaching them the academic skills they need to do good homework. The indirect effects model suggests that parental homework involvement affects children by promoting their motivation to engage with their homework and school tasks and do them well. According to this indirect or motivational model, when parents place importance on homework, children themselves come to value homework and develop the sense of competence that enables them to make efforts in learning activities, such as homework. Thus, the motivational homework model suggests that parental involvement in homework facilitates the motivational resources that enhance children’s homework engagement (Raftery et al., 2012). Both models would have similar explanations in the case of teacher involvement in homework.

### Teacher Involvement in Homework

Setting homework is an extremely widespread instructional practice (OECD, 2014). And, although the reasons for setting homework may be different depending on variables such as the type of culture (Moorhouse, 2018), teachers play absolutely critical roles in the homework process (Murillo and Martinez-Garrido, 2014). They play these important roles at two points in the homework process. In the first phase by setting the objectives of homework assignments and designing tasks; and in the final phase by implementing classroom follow-up practices (Cooper, 2001; Epstein and Van Voorhis, 2001; Rosário et al., 2015).

Some researchers found that middle and high school students who perceived their homework assignments as well-selected or well-prepared by their teachers reported higher motivation and effort at student and at class level (Trautwein and Lüdtke, 2007; Dettmers et al., 2010). As Trautwein et al. (2006) stated in their theoretical homework model, the perceptions of homework quality influence homework expectations and the value ascribed to it, which predicts homework effort. In a recent study with elementary students, Rosário et al. (2019) concluded that what seemed to explain achievement was the students’ perception of the quality of homework (i.e., assignments which are well-chosen by the teacher, which are interesting, related to the material taught in class, and useful for understanding the material covered in class), more than the type of homework set (see also, Fredricks, 2011).

Cunha et al. (2018a) explored teachers’ conceptions of homework feedback (47 teachers from elementary and middle schools participated in six focus groups) focusing on definition, purpose, types, and perceived impact. Teachers conceptualized homework feedback in three directions (i.e., teacher feedback to students, student feedback to teachers, and homework self-feedback). The most common purpose reported by most teachers was teacher monitoring of student learning, with checking homework completion and checking homework on the board being the most commonly used type of homework feedback in class. In another study at middle school level, Rosário et al. (2019) found that teachers’ purposes for homework follow-up practices in class were focused on identifying students’ learning strengths and weaknesses, promoting students’ engagement, and addressing students’ difficulties in mathematics. The follow-up practices included homework feedback provided in class: oral or written praise, criticism, written comments (highlighting right and wrong answers), rewards, general review of homework in class, and grading (e.g., Elawar and Corno, 1985; Corno, 2000; Cooper, 2001; Medwell and Wray, 2018).

These homework feedback practices are an important instructional tool for teachers in their teaching processes (e.g., helping identify students’ difficulties, errors or misconceptions in homework; approaching the learning content accommodating students’ lack of prior knowledge, and redesigning homework to match student needs) (Corno, 2000; Epstein and Van Voorhis, 2001; An and Wu, 2012). However, feedback provided by the teacher is also important for students because it is a way for the students to perceive the quality of their progress and help them to overcome difficulties they may have when doing homework (Trautwein et al., 2009; Núñez et al., 2015a). To be effective, feedback should provide information on the progress achieved and on how to act in the future. Providing feedback about a particular task should include information about how successfully it was done, providing an opportunity to improve and expand knowledge.

Previous research has shown that teacher homework feedback, as perceived by students, is positively related to student interest in homework (Xu, 2008), quality of student motivation toward homework (Katz et al., 2010), homework management strategies (Xu et al., 2017), and amount of homework completed and academic achievement (Núñez et al., 2015a). For example, the study by Núñez et al. (2015a), with students from various school years (grades 5–12), concluded that the better the student perception of teachers’ homework feedback, the greater the amount of homework completed and the better the homework time management. When students perceive their homework as of higher quality, they are more likely to put in more effort, complete homework more frequently, perform better on assignments, and achieve higher grades in mathematics. Moreover, these authors found that students’ academic achievement is indirectly and positively associated with teacher homework feedback through students’ homework behaviors and self-regulation (i.e., amount of homework completed; quality of homework time management), highlighting the importance of student engagement in the homework process. Research also shows engagement to be higher in students who have developed strong relationships with their teachers, in which the teachers support students’ autonomy, have high expectations, and give consistent and clear feedback.

### Parental Involvement in Homework

Patall et al. (2008) found positive effects in relation to parental involvement in homework, among other variables, in student attitudes to homework, and Pomerantz et al. (2007) found that parental behavioral involvement improves student achievement because it promotes student motivation and encourages student commitment. However, relationships between parental involvement in homework and academic achievement have been extensively debated and frequently researched (Gonida and Cortina, 2014; Gonida and Vauras, 2014) with inconsistent results. Some studies have found a positive relationship (e.g., Cooper et al., 2001; Pomerantz and Eaton, 2001), others have reported a negative relationship (Schultz, 1999), and others mixed results (e.g., Dumont et al., 2012).

In three longitudinal studies, Van Voorhis (2011) found a positive relationship between parental involvement, guided by a systematic intervention, and student achievement in mathematics, science, and language. Although some studies using structural equation models (SEM) have also reported a positive relationship between parental involvement and achievement (Cooper et al., 2001; Pomerantz and Eaton, 2001), others have found a negative relationship, and some, mixed results (Dumont et al., 2012). In particular, Dumont et al. (2012) found both positive and negative relationships depending on the quality of parental involvement and the different measures of the educational outcome (achievement, self-concept, and attitudes).

The mixed results may be due to multiple factors. Results vary depending on factors such as research design (Patall et al., 2008); content domain (e.g., subject-specific vs. general homework and academic achievement, Epstein and Van Voorhis, 2012); different dimensions of the construct measured (Dumont et al., 2012, 2013; Karbach et al., 2013); student school year (Cooper and Valentine, 2001), etc. Of all of those, the type of parental involvement may be one of the most determining factors (Ng et al., 2004; Pomerantz et al., 2007; Patall et al., 2008; Karbach et al., 2013; Gonida and Cortina, 2014; Suárez et al., 2014; Núñez et al., 2015b). Dumont et al. (2012, p. 64) suggested that “it is therefore crucial to distinguish between different dimensions of parental homework involvement and not to focus only on its quantity. Because different forms of parental homework involvement may have contrasting effects, an exclusive focus on the extent of parents’ involvement may lead to erroneous conclusions about its effectiveness.”

Different types of parental involvement in homework have been reported in the literature. For example, Hoover-Dempsey et al. (2001) describe eight ways in which parents can be involved in their children’s homework. From a more precise perspective, Pomerantz et al. (2007) indicated four qualitatively different dimensions of parent involvement in homework: autonomy support vs. control, process vs. person focus, positive vs. negative affect, and positive vs. negative beliefs about children’s potential. At a more systematic and operational level, Lorenz and Wild (2007), proposed four different types of parental involvement: autonomy supportive practices (i.e., parents encourage self-initiated homework activities), control (i.e., parents pressure children to complete their homework assignments and issue instructions that undermine autonomous behavior), structure (i.e., parents organize the homework environment), and emotional involvement (i.e., parents acknowledge children’s feelings about homework). Gonida and Cortina (2014), basing their work on various ideas from previous research, developed and validated a self-report scale that provides information directly through parents’ responses on four different forms of parental involvement in homework: (i) autonomy support and promotion of self-regulated learning, (ii) control, (iii) interference, and (iv) cognitive engagement related to schoolwork as supplementary to homework. Recently, Cunha et al. (2018b) validated the Parental Homework Management Scale (PHMS) for parents of elementary and junior high school children (ages 9–13 years) in the domain of mathematics, based on the responses of a sample of 2,118 parent–child dyads. The PHMS scale was originally constructed to measure four common types of parental involvement: (1) environment, (2) time, (3) motivation, and (4) emotion management. However, the results showed that at such early ages the PHMS is composed of two different but related factors: (1) environment-time management and (2) motivation-emotion management.

Different types of parental homework involvement have different implications for the student’s engagement with homework. Dumont et al. (2012) found both positive and negative relationships, depending on the nature or quality of the involvement. For example, whereas perceived parent–child conflicts about homework were negatively associated with educational outcomes, perceived parental competence and support for students’ self-direction were positively related to achievement. Similar results were reported by Karbach et al. (2013), who found that academic achievement was significantly and negatively associated with parental control and strict structure (i.e., excessive control and pressure on children to complete assignments, consistent guidelines and rules about homework and school work). Gonida and Cortina (2014) saw different patterns of student gain depending on the type of parental involvement in homework: autonomy support was the most positive (parents who are involved giving support and favoring the autonomy of the child promote the development of a motivational orientation directed to learning and mastery), while interference was the most damaging (because it undermines mastery goal orientation and reduces perceived competence). Data from the study by Cunha et al. (2018b) showed a similar picture to that of previous studies (e.g., Dumont et al., 2012; Karbach et al., 2013; Gonida and Cortina, 2014): the two dimensions of the PHMS (i.e., environment-time and motivation-emotion management) were positively associated with homework self-regulation strategies and positive homework emotions. Finally, Silinskas and Kikas (2019) found that perceived parental control negatively and significantly predicts mathematical performance, student self-concept and student persistence. However, perceived parental support positively predicts student task persistence.

So, the results from past research show without a doubt that autonomy support is the most advisable form of parental involvement in children’s homework. Parental homework autonomy support can encourage the development of intrinsic motivation toward homework (see also Katz et al., 2011; Madjar et al., 2016; Moè et al., 2018; Feng et al., 2019), increased perceived competence and homework management (Xu et al., 2017; Moè et al., 2018), and task persistence (Silinskas and Kikas, 2019), as well as reducing procrastination (Katz et al., 2014). In general, all of this suggests that parental homework involvement may play a valuable role in student homework management.

### Role of Student Age and Gender

The association between parental homework involvement and student achievement proved to be mediated by school year (Skaliotis, 2010), happening less frequently as students grow older (Hoover-Dempsey and Sandler, 1997), although the data we have available seem to suggest greater consistency in middle and high school than in elementary school (see Chen, 2008; Patall et al., 2008). Silinskas and Kikas (2019) reported mixed results from their study with elementary school students. On the one hand, the results showed that perceived parental support was positively related to student task persistence, but the relationship disappeared when the sample was split by gender. Differences related to school year in the relationship between parental homework involvement and student homework management were also found by Núñez et al. (2015b). The data from that study indicated that perceived parental homework support and control was not related to student homework behaviors at the elementary school level, there was considerable association at the junior high school level, and more targeted association at the high school level. Finally, the study by Gonida and Cortina (2014), found differences associated with school year (elementary and junior high school years) in parental homework involvement. However, those differences were related to the mean scores for some of the variables (i.e., parent autonomy homework support and control), but no differences were seen in the structural part of the full mediation model tested.

Findings from Núñez et al. (2015b) suggested that higher school years (Grades 5–12) were associated with lower levels of perceived homework feedback from teachers. This coincides with data from other studies (e.g., Katz et al., 2010).

With respect to student variables (homework motivation and homework engagement), the available data suggest that as students move from elementary to high school, motivation and engagement decrease. For example, Katz et al. (2010) found school-year-related differences in student homework autonomous motivation: junior high school students have lower motivation than elementary school students. Similar data has been seen in studies carried out in different cultures and environments. Hong et al. (2009), analyzing Chinese students’ (7th and 11th graders), concluded that older students were less engaged, persisted less, and expressed less enjoyment doing homework than younger students did. This pattern of devaluing school work, and exhibiting less effort and persistence when completing homework is in line with other studies and analyses in western cultures (e.g., Epstein and Van Voorhis, 2012; Regueiro et al., 2018). The data from samples of European students gives us the same picture: statistically significant differences as a function of school year in student homework motivation and engagement. For example, the study from Regueiro et al. (2015) with fourth to tenth grade students found that students in the higher grades, compared with the youngest, are less interested in homework, find it less useful, and have a more negative attitude toward homework.

Finally, several studies have looked at gender. For example, in a recent study, Madjar et al. (2016) did not find statistically significant differences in boys and girls in goal orientation toward homework, although Xu (2006) had found such differences. In middle school students, Feng et al. (2019) found that boys reported higher homework autonomy motivation than girls. On the other hand, in contrast to the data from Xu and Corno (2006), Núñez et al. (2015a), reported the absence of gender differences in the perception of teacher homework feedback.

### The Current Study: Goals and Hypotheses

In this study we intend to analyze the validity of the indirect effects model (or motivational model) of student homework engagement, in students from two different school levels (middle and junior high school students), and by gender. We will analyze the extent to which motivation mediates the effect of the involvement of parents and teachers on student homework engagement (i.e., use of SRL strategies). In general terms, we intend to test the hypothesis that students’ autonomous motivation to do quality homework mediates the relationship between perceived teacher and parental involvement on homework and the students’ homework engagement. For a mediating effect to occur, the mediator variable must be significantly related to both the independent variable and the dependent variable. Based on the results of previous research (e.g., Katz et al., 2010; Feng et al., 2019), which support a model of indirect effects or motivational model (Raftery et al., 2012), in this study we hypothesize that (i) student’s perceptions of the involvement of their parents and teachers in their homework significantly influences their motivation toward homework, and (ii) that this in turn influences their engagement (Bouffard et al., 2001) in the realization of quality homework (i.e., use of self-regulated learning strategies in homework).

**FIGURE 1:**
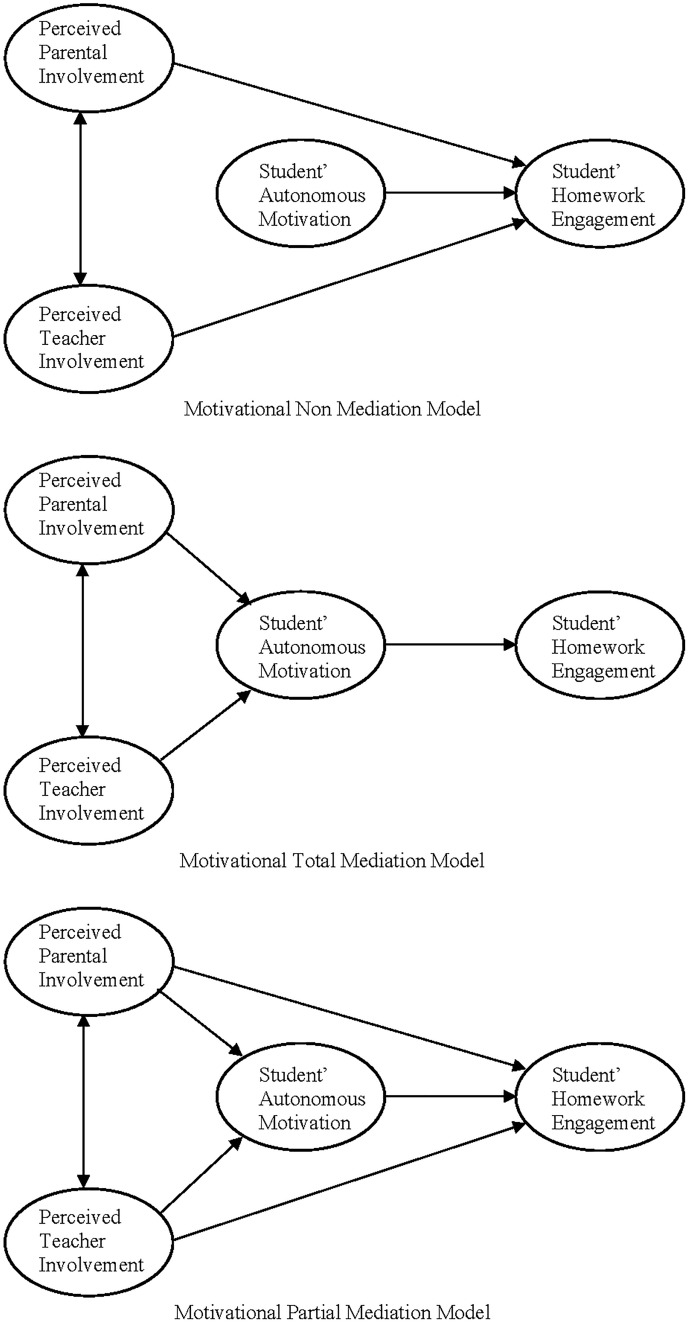
Three versions of the motivational mediation model of homework (non, total and partial mediation).

Data from previous research lead us to specify both hypotheses in the following terms (see Figure 1). First, we expect that the perception of involvement of both parents and teachers in homework will significantly and positively affect student homework motivation. The greater the perception of the involvement of teachers and parents in homework, the more motivated the student, and vice versa (e.g., Epstein and Van Voorhis, 2001; Patall et al., 2008; Karbach et al., 2013; Núñez et al., 2015a,b, 2017; Rosário et al., 2015, 2018). However, given the more direct relationship between teachers and homework, as reported in other studies (e.g., Feng et al., 2019), we expect teachers’ behavior to be a more powerful predictor than parents’ behavior. Secondly, we also expect the use of self-regulation strategies for working on homework to be significantly and positively conditioned by student’s motivation for homework engagement (e.g., Midgley, 2002; Vansteenkiste et al., 2005; Valle et al., 2015). Students who are more motivated toward the task (with the intention of learning) will tend to use more self-regulation strategies in their homework than students with less task-oriented motivation.

In order to examine these hypotheses, we formulated a model of structural equations with three variants: (i) a no motivational mediation model, or the direct effects model; (ii) a total motivational mediation model, or indirect effects model; and (iii) a partial motivational mediation model, or mixed effects model. In the total motivational mediation model, the effect of the perception of involvement of parents and teachers in homework on the use of self-regulation learning strategies occurs entirely through student homework autonomous motivation (there is an indirect effect, but not direct). However, there is partial mediation when, at the same time, both an indirect (mediation) and a direct effect occur. Finally, non-mediation takes place when the perception of involvement of parents and teachers in homework is not related to the mediating variable (i.e., homework autonomous motivation), and instead they directly influence the use of self-regulated learning strategies when working on homework.

Previous research leads us to assume a total motivational mediation hypothesis (although partial mediation could also occur). According to a model of total motivational mediation (see Figure 1), we hypothesize that:

H1: Perceived parental involvement (i.e., parental content-oriented support) has a positive and statistically significant effect on student’s motivational involvement in homework (i.e., homework autonomous motivation), but not on student’s homework engagement (i.e., student homework engagement).
H2: Perceived teacher involvement (i.e., teachers’ homework management) has a positive and statistically significant effect on student’s motivational involvement in homework (i.e., homework autonomous motivation), but not on student’s homework engagement (i.e., student homework engagement).
H3: Student’s motivational homework involvement (i.e., homework autonomous motivation) is positively and statistically significantly related to subsequent homework engagement (i.e., student homework engagement).
H4: Perceived teachers homework involvement (i.e., teachers’ homework management) determines students’ motivational involvement in their homework (i.e., homework autonomous motivation) to a greater extent than perceived parents’ involvement (i.e., parental content-oriented support).
H5: Taking the results of the study from Gonida and Cortina (2014) as a reference, we expect no significant differences in the homework motivational model (structural part of the model) between boys and girls.
H6: In relation to school year, although this model has not been tested at different ages (the study by Feng et al., 2019, only used middle school students), based on the data provided by other researchers that have worked with different academic levels with respect to effects of parental involvement (e.g., Cooper and Valentine, 2001; Núñez et al., 2015b) and teacher involvement (e.g., Trautwein and Lüdtke, 2007; Katz et al., 2010; Xu et al., 2017) in students’ homework, we hypothesize the existence of statistically significant differences in the structural part of the established model.

## Materials and Methods

### Participants

The sample included 730 students in 4 years of Compulsory Secondary Education (CSE) in Spain who were enrolled in one of the 14 public schools participating in the study (located in three provinces in northern Spain). Approximately half of the schools are located in urban areas, and the other half are in rural or semi-urban areas. Just over half (56.6%) of the students were girls. The distribution of participants by year is similar: 26.6% in 7th grade; 20.8% in 8th grade; 24.9% in 9th grade; and 27.7% in 10th grade. The ages of the participants ranged between 12 and 16 years old.

### Instruments

The variables Perceived Parental Homework Involvement and Perceived Teacher Homework Involvement were obtained from various items of the *Homework Survey* (see Appendix), used in previous research (e.g., Núñez et al., 2015a,b; Valle et al., 2015).

#### Perceived Parental Homework Involvement (PPHWI)

This measures parents’ supportive behavior (as perceived by the students) when their children do homework (see Appendix). The three items in this subscale were taken from the Parental Homework Support Scale (Xu et al., 2017). The measure mainly has to do with perceived parental content-oriented support, rather than parental homework autonomy as such. The students’ responses are rated on a five-point Likert-type scale ranging from 1 (*totally false*) to 5 (*absolutely true*). Taking into account the small number of items (three), the measure shows good reliability in the current study (α = 0.84).

#### Perceived Teacher Homework Involvement (PTHWI)

This evaluates the teacher’s feedback perceived by students when the students do homework in the classroom (see Appendix). It requests information about teachers’ behavior in adapting homework to students’ difficulties and supervising their level of comprehension, as well as errors made. In this study it is understood in the sense of teacher homework management (homework handling). Responses are rated on a 5-point Likert-type scale ranging from 1 (*totally false*) to 5 (*absolutely true*). Although the number of items is small (three), the reliability of the measurement in this study is moderate (α = 0.60).

The variables Student Homework Autonomous Motivation and Student Homework Engagement were provided by the students’ responses to the “Inventario de Procesos de Estudio” [(Study Process Inventory) Rosário et al., 2013], after adapting it to the process of doing homework. Respondents rate each one of the six items on a five-point Likert-type scale ranging from 1 (*totally false*) to 5 (*absolutely true*).

#### Student Homework Autonomous Motivation (SHWAM)

We estimated students’ greater or lesser motivational involvement through their intention to master the homework and learn (task orientation). This instrument evaluates students’ interest in homework, their satisfaction when doing it, as well as their positive feelings about working that way. The three items offer a measure of students’ autonomous motivation for homework similar to that provided by the scale from Katz et al. (2011) and used in Moè et al. (2018). Taking into account the small number of items (three), the reliability of the measurement in this study is acceptable (α = 0.73).

#### Student Homework Engagement (SHWE)

Students’ engagement in homework was measured with three items that asked them about the self-regulated learning strategies used when doing homework (planning, monitoring, and evaluation). The three items were extracted from the Self-Regulation Learning Strategies Inventory (Rosário et al., 2012), and provide information about the use of a macro-SRL strategy consisting of the three phases described by Zimmerman’s Model (e.g., Zimmerman, 2011). The reliability of the measure is modest in this study (α = 0.70), but if we take into account the number of items (three), it can be considered acceptable.

### Procedure

The target variable data were collected during regular school hours, after obtaining the consent of the school directors and the students’ parents. The questionnaires were administered in a single session by specialized staff that collaborated in the investigation. Participants completed all the questionnaires individually and without a time limit. The procedures followed in the study were in accordance with the ethical standards of the Research and Teaching Ethics Committee of the University of A Coruña, the University of Oviedo, and the Helsinki Declaration.

### Data Analysis

The structural equation model (SEM) was adjusted with the AMOS 22 program in SPSS (Arbuckle, 2013). Students with a large number of missing values were removed from the database (1.23%), while the rest of the missing values were imputed.

The data were analyzed in three steps. Firstly, we calculated and reviewed the descriptive statistics and the Pearson correlation matrix. Secondly, considering that the variables exhibited a normal distribution, we estimated the goodness of fit of three versions of the structural equation model using robust maximum likelihood (RML): (i) no mediation (direct effects model), (ii) total mediation (indirect effects model), and (iii) partial mediation (mixed effects model). Thirdly, based on the AIC and BIC statistics, the best model of the three was identified and adjusted for the total population, for boys and girls, and for the two school levels. While we initially used data from 4 years (7th–10th grade) for the analysis of the effect of this variable, we regrouped the 4 years into two groups: middle school (7th and 8th grade) and junior high school (9th and 10th grade). In the Spanish educational system, 7th and 8th grade correspond to the first cycle of CSE and 9th and 10th grade to the second cycle of CSE. In the Spanish system these educational stages have different motivational, affective, cognitive and behavioral requirements at the student and context level. The first stage (7–8th) in which students “feel older” (e.g., greater autonomy, less parental control) also involves difficulties in adapting to a very different situation from the one they have left behind (new classmates, new friends, new teachers, etc.), and is more demanding. In contrast, in the second stage (9–10th) the students are more confident (of themselves and of the context), and have greater perceived control. Similarly, parents and teachers expect more autonomy from them but also more responsibility. This second stage also represents the end point of compulsory education. Taking all that into account, it seemed appropriate to adjust the homework motivational model in the two stages separately.

Model fit was evaluated using the most important indexes and statistics from AMOS 22 [i.e., χ^2^, χ^2^/*df*, the adjusted goodness-of-fit index (AGFI), the comparative fit index (CFI), and the root mean square error of approximation (RMSEA)]. Evidence of a good fit is when χ^2^ has a *p* > 0.05, χ^2^/*df* < 5, AGFI ≥ 0.90, CFI ≥ 0.95, and RMSEA ≤ 0.05. The smallest values of AIC and BIC indicate the best model. The effect size of the regression coefficients were calculated using Cohen’s (1988) *d* statistic.

## Results

Following the data analysis strategy above, the results are described in three sections: (a) descriptive statistics; (b) selecting the best model; and (c) fit of the selected model and parameter evaluation.

### Descriptive Statistics

Table 1 gives the descriptive statistics for the total sample of students. The data show: (1) that the variables were significantly correlated with each other (all the correlation coefficients were statistically significant), and (2) that the symmetry and kurtosis of the variables indicated a sufficiently normal distribution.

**Table 1:** Pearson correlations, mean, standard deviation, skewnes, and kurtosis of observed measures.

	PPHWI1	PPHWI2	PPHWI3	PTHWI1	PTHWI2	PTHWI3	SAM1	SAM2	SAM3	SHWE1	SHWE2	SHWE3
PPHWI1	–											
PPHWI2	0.604	–										
PPHWI3	0.584	0.704	–									
PTHWI1	0.216	0.201	0.234	–								
PTHWI2	0.130	0.162	0.178	0.354	–							
PTHWI3	0.187	0.201	0.219	0.418	0.251	–						
SAM1	0.182	0.166	0.216	0.296	0.185	0.275	–					
SAM2	0.171	0.192	0.227	0.262	0.200	0.278	0.563	–				
SAM3	0.210	0.230	0.319	0.243	0.196	0.180	0.478	0.401	–			
SHWE1	0.192	0.196	0.268	0.321	0.167	0.257	0.531	0.535	0.424	–		
SHWE2	0.227	0.214	0.254	0.242	0.178	0.180	0.390	0.338	0.427	0.414	–	
SHWE3	0.223	0.208	0.209	0.233	0.155	0.237	0.446	0.402	0.434	0.476	0.434	–
M	3.17	3.21	3.21	3.50	2.07	4.20	3.28	3.69	2.45	3.29	2.63	2.89
SD	1.46	1.52	1.43	1.24	1.24	0.98	1.20	1.13	1.26	1.15	1.19	1.14
Skewness	-0.21	-0.21	-0.21	-0.50	0.92	-1.15	-0.26	-0.63	0.47	-0.30	0.28	0.04
Kurtosis	1.46	1.52	1.43	1.24	1.24	0.98	1.20	1.13	1.26	1.15	1.19	1.14


*PPHWI1 to PPHWI3 are observed measures of Perceived Parental Homework Involvement; PTHWI1 to PTHWI3 are observed measures of Perceived Teacher Homework Involvement; SAM1 to SAM3 are observed measures of Student Autonomous Motivation; SHWE1 to SHWE3 are observed measures of Student Homework Engagement. All variables have the same scale: minimum = 1, maximum = 5. All correlation coefficients are statistically significant at p ≤ 0.01.*

### Selecting the Best Model

Table 2 shows the results of the adjustment of the three models in competition. The data indicates that the two models that include mediation (total and partial mediation models in Figure 1) have excellent indexes of fit. The small difference in the values of the fit indexes of the two models is due to the fact that the two direct effects that made partial mediation possible (perceived involvement of parents and teachers on the student’s engagement in homework) are not statistically significant (perceived parental involvement → student homework engagement = 0.061, *p* > 0.05; perceived teacher involvement → student homework engagement = 0.036, *p* > 0.05). However, because the AIC and BIC values of the total mediation model are lower than those of the partial mediation model, and because the total mediation model is more parsimonious than the partial mediation model, we selected the total mediation model as the model with best fit.

**Table 2:** Results of the fit of the three competing motivational mediation models.

	Non-mediation model (NMM) (Direct effects model)	Partial mediation model (PMM) (Mixed effects model)	Total mediation model (TMM) (Indirect effects model)
χ^2^ (*df*)	255.398 (50)	103.963 (48)	106.847 (50)
*p*	<0.001	<0.001	<0.001
χ^2^/*df*	5.108	2.166	2.137
AGFI	0.918	0.961	0.961
CFI	0.926	0.980	0.980
RMSEA [LO, HO]	0.075 [0.066, 0.084]	0.040 [0.029, 0.051]	0.039 [0.029, 0.050]
AIC	311.398	163.963	162.847
BIC	440.003	301.754	291.452


### Evaluation of the Total Mediation Model of Homework

The Total Mediation Model was adjusted for the total sample, for boys and girls, and for school years, grouped into two levels [middle (7th–8th) and junior high school (9th–10th)]. Table 3 presents the corresponding fit statistics. The data show an excellent fit of the model in all cases for the total sample and for the four specific samples. These results suggest that the Total Mediation Model does not require additional modifications.

**Table 3:** Goodness-of-fit statistics for the Motivational Total Mediation Model of homework in the overall sample, and by gender and grade.

Sample	χ^2^(*p*)	χ^2^/*df*	AGFI	CFI	RMSEA [LO, HO]
Total	106.85 (0.000)	2.139	0.96	0.98	0.039 [0.029, 0.050]
Girls	94.45 (0.000)	1.889	0.94	0.97	0.048 [0.033, 0.063]
Boys	60.70 (0.143)	1.214	0.95	0.99	0.027 [0.000, 0.047]
7th–8th grade	63.03 (0.102)	1.261	0.95	0.99	0.028 [0.000, 0.048]
9th–10th grade	84.75 (0.002)	1.695	0.94	0.97	0.043 [0.026, 0.058]


*The models have 50 degrees of freedom*.

Table 4 shows the standardized regression coefficients, statistical significance, and effect size corresponding to the fit of the model in the four specific samples and in the total sample. In general, the data support the motivational total mediation model, both for girls and boys and for the two school levels analyzed. The data in Table 4, relative to the total sample give good support to the hypotheses that produce the motivational model of total mediation.

**Table 4:** Standardized regression coefficients of the Motivational Total Mediation Model of homework.

Standardized direct effects	Standardized regression weights	Standard error	Critical ratio	Probability *P<*	Effect size *d*
Total sample					
PPHWI → SAM	0.192	0.041	4.038	<0.000	0.302
PTHWI → SAM	0.501	0.065	7.860	<0.000	0.608
SAM → SHWE	0.953	0.054	16.913	<0.000	1.605
PPHWI ↔ PTHWI	0.399	0.051	7.096	<0.000	0.544
Gender samples					
Females (*n* = 383)					
PPHWI → SAM	0.250	0.050	4.001	<0.000	0.417
PTHWI → SAM	0.461	0.071	5.701	<0.000	0.536
SAM → SHWE	0.950	0.072	12.450	<0.000	1.649
PPHWI ↔ PTHWI	0.331	0.071	4.652	<0.000	0.489
Males (*n* = 294)					
PPHWI → SAM	0.116	0.076	1.429	0.153	0.167
PTHWI → SAM	0.501	0.126	4.659	<0.000	0.564
SAM → SHWE	0.942	0.084	10.278	<0.000	1.497
PPHWI ↔ PTHWI	0.449	0.079	4.678	<0.000	0.567
Grade samples					
7th–8th (*n* = 346)					
PPHWI → SAM	0.129	0.065	1.724	0.085	0.186
PTHWI → SAM	0.596	0.164	4.965	<0.000	0.554
SAM → SHWE	0.951	0.078	12.150	<0.000	1.725
PPHWI ↔ PTHWI	0.386	0.060	4.202	<0.000	0.463
9th–10th (*n* = 384)					
PPHWI → SAM	0.212	0.057	3.345	<0.000	0.346
PTHWI → SAM	0.402	0.077	4.927	<0.000	0.519
SAM → SHWE	0.886	0.076	10.730	<0.000	1.308
PPHWI ↔PTHWI	0.272	0.067	3.782	<0.000	0.393


*PPHWI (Perceived Parental Homework Involvement), PTHWI (Perceived Teacher Homework Involvement), SAM (Student Autonomous Motivation), SHWE (Student Homework Engagement). Effect (→), relationship (↔).*

H3 was confirmed: motivation was a powerful determinant of the use of self-regulated learning strategies doing homework (student homework engagement), both in the total sample and as a function of gender and school level (the regression coefficients were higher than *b* = 0.90), except for the junior high school sample (9th–10th grade), which was slightly lower (*b* = 0.89). The effect sizes were very large (see the *d* statistic in Table 4). Likewise, H1 and H2 were confirmed in the total sample. The data also support the hypothesis about the association between perceived teacher involvement on homework and student autonomous motivation, with a moderate effect size (*d* = 0.608), and the hypothesis about the relationship between perceived parental involvement in homework and student autonomous motivation, which was significant, albeit with a small effect size (*d* = 0.302). Confirming the fourth hypothesis (H4), that perceived teacher involvement in homework has a greater relationship than perceived parental involvement in homework with students’ autonomous homework motivation. The confirmation of the first three hypotheses (along with the fourth) allow us to conclude that student autonomous motivation mediates the relationship between the involvement of parents and teachers perceived by students and student homework engagement.

Student homework engagement is explained to large degree (90.9%) by the direct effect of student autonomous motivation, but also due to the indirect effect of perceived teacher and parent homework involvement through student autonomous motivation. More specifically, of the total explanation of student homework engagement, the unique effect of student autonomous motivation is 24.79%; the effect corresponding to perceived parental homework involvement on student homework engagement through student autonomous motivation is 18.29%; and the effect corresponding to perceived teacher homework involvement on student homework engagement through student autonomous motivation is 47.74%. As it is a model of total mediation of student motivation, the direct effect of teacher and parent on student homework engagement is zero. Finally, parent and teacher homework involvement explain 36.4% of student autonomous motivation, directly (28.78%: 3.68% parents and 25.10% teachers) and indirectly (7.62%; one through the other: *r* = 0.399, *d* = 0.870).

The data support only a partial confirmation of the fifth hypothesis (H5). There are no significant differences in terms of two of the three direct effects of the model: both girls’ and boys’ perception of teacher homework involvement is statistically and significantly related to student autonomous motivation, to a similar extent (with a moderate effect size, slightly higher than *d* = 0.50). On the other hand, girls and boys exhibit positive, statistically significant and similar relationships between student autonomous motivation and student homework engagement (with a very large effect size, around *d* = 1.5). However, there are differences between the two groups in the association between perceived parental homework involvement and student autonomous motivation: while it is positive and statistically significant for girls (with a moderate effect size), it is not statistically significant in the sample of boys. Therefore, the data suggest that in the sample of girls there is mediation of student autonomous motivation in the relationship between perceived parental homework involvement and student homework engagement, while this is not so in the sample of boys. In other words, boys’ homework engagement is not explained by perceived parental homework involvement.

Finally, the data related to school year (H6), indicate that the relationship between student autonomous motivation and student homework engagement does not vary according to whether the students are in middle or junior high school. Likewise, they are not significantly different in the association between perceived teacher homework involvement and student autonomous motivation. However, as with gender, significant differences were found in the association between perceived parental homework involvement and student autonomous motivation. In particular, while the relationship is statistically significant in junior high school (although the effect size is small, *d* = 0.346), it is not in middle school (*p* > 0.05). Also in this case, the data suggest that there is no mediation at middle school: perceived parental homework involvement does not directly or indirectly determine student homework engagement.

### Ancillary Analyses

The data in Table 4 indicate that the association between student autonomous motivation and student homework engagement is very strong, both for the total sample (*b* = 0.953) and for girls (*b* = 0.950), boys (*b* = 0.942), middle school students (*b* = 0.951), and junior high school students (*b* = 0.886). This adds fuel to the fire of the dispute over whether they are similar or different constructs. Although in this study we have assumed the theoretical position that motivation and engagement are different constructs, and were treated that way in the formulation of the model and the treatment of the data, there is no doubt that the two variables are intimately related, as the aforementioned data demonstrates. Are they different constructs (e.g., Russell et al., 2004; Reeve, 2012) or are they two dimensions of a macro-construct (e.g., Fredricks et al., 2004; Martin, 2007)?

To answer this question, we produced two models by confirmatory factor analysis, with one and two factors, taking observed measures as the answers to the three items that theoretically measure student homework autonomous motivation and the three that theoretically measure student homework engagement. If the unifactorial model has the best fit, we could say we are faced with a macro-construction where motivation and engagement are two sides of the same coin. However, if the bifactorial model offers the best fit, then we may conclude that these are related but different constructs.

The data provided by the CFA seems to support a two-factor model. Although the fit of both models is good [one factor model: χ^2^_(9)_ = 48.014, *p* < 0.001, GFI = 0.977, AGFI = 0.946, TLI = 0.950, CFI = 0.971, RMR = 0.046, RMSEA = 0.078; two factor model: χ^2^_(8)_ = 41.455, *p* < 0.001, GFI = 0.980, AGFI = 0.946, TLI = 0.954, CFI = 0.980, RMR = 0.045, RMSEA = 0.076], the two factor model fits significantly better than the one factor model since the AIC is smaller (AIC one factor model = 72.014; AIC two factor model = 67.455). Therefore, the data seem to suggest that student motivation and student engagement are closely related but distinct constructs. The results of this research do not solve the question at all, so it may be a good idea to design a highly controlled study with zero threats to the validity.

## Discussion

In this study, we wanted to analyze the mediating role of students’ autonomous homework motivation in the relationship between perceived parental and teacher involvement in homework and the students’ homework engagement (i.e., use of SRL strategies in homework). In order to examine this hypothesis, we produced a structural equation model, and three versions (no motivational mediation, partial motivational mediation and total motivational mediation) were tested, for the total sample, and by gender and school year (middle and junior high school). Below, we discuss the results and their educational implications. We also describe some limitations of the study that could influence the data.

From a general point of view, the data suggest a total motivational mediation model, with some differences by gender and by school year. Despite the differences, we can conclude that motivation completely mediates the effect of teacher and parental involvement on students’ homework engagement (i.e., the use of self-regulated learning strategies).

The results of this study are largely in line with those from Feng et al. (2019), in that autonomous motivation mediates the relationship between perceived teacher homework management and perceived parent homework content-oriented support and student homework engagement. However, in our study, autonomous motivation mediated completely between perceived parent content-oriented support and student homework engagement, whereas the study by Feng et al. (2019) reported partial mediation.

As in previous research (e.g., Valle et al., 2016), in this study students’ homework engagement is directly predicted by student autonomous motivational engagement (interest in learning and/or gaining competence and autonomy). As in other studies (e.g., Midgley, 2002; Vansteenkiste et al., 2005; Valle et al., 2015; Veas et al., 2018), the results suggest that student engagement in homework depends greatly on being motivated to acquire competence and autonomy. However, the dependence of autonomous motivation and student engagement in our study is even stronger than in previous studies. Our data seem to suggest that the three variables considered as predictors of student homework engagement really are predictors, and do not vary by gender or student age. Our data from secondary education students (7th–10th grade) complement the data from Valle et al. (2016), although that was from students in 4th, 5th, and 6th grades.

Likewise, the results in this study about the relationship between parent and teacher homework involvement and student homework autonomous motivation are in accordance with the initially proposed hypotheses in the case of the total sample, but not when gender or school year are considered.

More specifically, when it comes to parents’ involvement in their children’s homework (i.e., content-oriented support), in line with other studies (e.g., Epstein and Van Voorhis, 2001; Van Voorhis, 2001; Pomerantz et al., 2007; Patall et al., 2008; Karbach et al., 2013; Gonida and Cortina, 2014; Gonida and Vauras, 2014; Suárez et al., 2014; Núñez et al., 2015b; Cunha et al., 2018b; Moè et al., 2018; Feng et al., 2019; Silinskas and Kikas, 2019), when children perceive that their parents provide support (i.e., oriented to content), their interest grows due to increased competence and autonomy through their engagement in homework. However, the size of this association is weaker than expected. Although some studies have reported a moderate effect size (e.g., Katz et al., 2011; Moè et al., 2018; Feng et al., 2019), the data from our study, without looking at student age or gender, have a modest (e.g., Gonida and Cortina, 2014) to small (e.g., Silinskas and Kikas, 2019) effect size.

Looking at the responses of 5th and 8th grade students, Gonida and Cortina (2014) found a positive relationship between parent autonomy and student mastery (*b* = 0.18, *p* < 0.01), with a modest effect size. Despite finding differences between 5th and 8th grade in mean scores for some of the latent variables, they found no differences in the relationship between the variables. However, as in the study from Silinskas and Kikas (2019), in our study we also saw differences between girls and boys in the effect of perceived parental content-oriented support on student autonomous motivation. In addition, our study also found a link between middle and junior high grades. In terms of gender, the size of the effect of girls’ perceptions of parental content-oriented homework support on their autonomous motivation toward homework is moderate, in boys this relationship is not statistically significant. In other words, the girls’ autonomous motivation for homework is much more sensitive to variations in the perception they have of the involvement of their parents in homework than in boys. In terms of age, our data indicate that the effect of perceived parental content-oriented support on student autonomous motivation is higher in junior high school (although the effect size is small) than in middle-school (the size of the effect is null). If we combine these results with those from other investigations in which parent homework support and student autonomous motivation for homework was seen to decrease as students age (e.g., Hong et al., 2009; Katz et al., 2010; Gonida and Cortina, 2014; Núñez et al., 2015b; Regueiro et al., 2018), the result seems somewhat paradoxical. As less student autonomous motivation is reported and less parental content-oriented support is perceived as the student gets older, the greater the impact of perceived parental content-oriented support on student autonomous motivation for homework. In other words, as one goes from 7th to 10th grade, there is less autonomous motivation for homework, lower perceived parental content-oriented support but nevertheless, a stronger relationship between the two variables (i.e., student autonomous motivation depends more on perceived parental content-oriented support). The explanation could lie in the child’s own development. It is possible that this happens because as the child grows in competences (cognitive, motivational and affective) they find it logical for their parents to require them to be more autonomous while at the same time they have a better understanding of the importance of their parents’ involvement in their homework.

The data on the effect of perceived teacher homework management on student autonomous motivation were completely in accordance with the hypothesis, both with and without controlling for gender and age. These results are consistent with previous research (e.g., Cooper, 2001; Epstein and Van Voorhis, 2001; Trautwein and Lüdtke, 2007; Trautwein et al., 2009; Dettmers et al., 2010; Katz et al., 2010; Xu et al., 2017; Feng et al., 2019), highlighting the important role of the association between teacher involvement in homework (e.g., feedback, follow-up practices, and designing homework) and student homework engagement (e.g., homework management strategies, time spent, amount of homework completed, and homework effort), and disengagement (Bempechat and Shernoff, 2012).

The impact of perceived teacher homework management on student autonomous motivation in our study is rather significant (with a moderate effect size in all cases), both in middle and junior high school, although perceived teacher content-oriented support decreases as students get older, both in our study [*t*_(728)_ = 9.441; *p* < 0.001; medium effect, *d* = 0.70] and in previous studies (e.g., Katz et al., 2010; Núñez et al., 2015a). We believe that both results have a reasonable explanation. Perhaps the decrease in student perceptions of teacher involvement in homework management as they go up the grades may be a true reflection of what actually happens (as students get older, teachers support more student autonomy). And in relation to the effect of perceived teacher homework management on student homework management, it is well understood that the strength of the association is maintained, since in both middle and junior high school it can be equally important for students to perceive that their teachers (i) make sure students understand the assigned tasks, (ii) consider the students when deciding the type of homework, or difficulty and (iii) what homework they see in class to correct mistakes. This seems to be an acceptable explanation for the similar effect sizes in girls and boys.

Although in this study there were gender differences in the mean scores of perceived teacher homework management in favor of girls [*t*_(675)_ = 2.90, *p* < 0.01, small effect size: *d* = 0.22], gender was not a factor related to the intensity of the effect of perceived teacher homework management on student homework engagement (a very similar effect size, see Table 4). This suggests that the autonomous motivation for homework is equally affected by perceived teacher homework management in boys and girls.

### Limitations of the Study

The study has some limitations which must be taken into account in the interpretation of the results, comparison with other studies, and generalization to other educational levels, contexts or cultures. Three are particularly important.

Firstly, the measures used to construct the latent variables of the homework motivational model were taken only through self-report scales. The importance of self-report methodology in educational research is undeniable (Zimmerman, 2011), but so are the associated problems of validity and reliability (Pike and Kuh, 2005), and incongruence with other innovative methods of assessment (Winne and Perry, 2000; Azevedo et al., 2017). In addition, in this research only three items per variable were used, which could be associated with some of the problems we indicated. For example, the internal consistency of three of the four scales is within the limits of what is acceptable (i.e., perceived teacher homework involvement, student homework autonomous motivation, and student homework engagement). Likewise, three items may be too few to adequately capture everything we wanted to measure. This is the case, for example, of the measure of perceived teacher homework management: three items are used that purport to provide information on three types of teacher actions that, while undoubtedly important, may not cover the construct “teacher homework management.”

Secondly, the measures in this study regarding the involvement of parents and teachers correspond only to the perception of the students (i.e., parental homework involvement and teacher homework involvement perceived by the students). We were interested in the perception of the student, more so than that of the teacher or the parents. Although the literature supports the need to consider students’ perspectives of homework assignments (e.g., Warton, 2001; Landers, 2013) because students are active players in their learning process (e.g., Trautwein and Lüdtke, 2007), it also recognizes the advantage of collecting and combining reports from different data sources (e.g., Dettmers et al., 2010; Saban, 2013; Rosário et al., 2018). However, in this study only the perception of the students was included, due to the weak relationship seen in other studies between the perception of the student and the perceptions of teachers or parents (i.e., Rosário et al., 2018). It is important to underline this in order for it not to be ignored if the data from this study were to be used in future studies, such as meta-analyses.

Another limitation is that the study is a cross-sectional survey. The data do not support causal analysis, even though our interpretations are based on previous findings and theoretical analysis. This issue must be addressed by future research, through repeated measure designs (e.g., Silinskas et al., 2013; Silinskas and Kikas, 2019) or using experimental or quasi-experimental designs which are as ecologically valid as possible (e.g., study 2 from Moè et al., 2018).

### Educational Implications

This study has clear educational implications. First, we found that one of the most important predictors of student homework engagement is student autonomous motivation (directly) and teacher and parental homework involvement (indirectly, through autonomous motivation). This highlights the importance of parents and teachers focusing on making students see that doing homework is not a punishment, or wasted time, but an opportunity to gain competence and above all, autonomy (Pomerantz et al., 2007).

Student engagement is affected by parents not only in terms of how much they participate, but also through the style with which they relate to their children in school-related tasks and aspects (Grolnick et al., 1997), including homework (Pomerantz et al., 2007). Parental autonomy support has significant consequences for motivation and student homework engagement. However, the impact should be greater than that reported by research. Because of this, it seems urgent to design interventions with parents in order to work with them to effectively use am autonomy support parental style when helping their children with homework. It is possible that this type of training would make this behavior clearer and more visible in the eyes of their children (as with the control style). An example of this type of intervention can be seen in Moè et al. (2018). The data from Moè’s study showed that the training program reduced parental negative affect, and prevented a decrease in student homework motivation and emotions.

The effect of teacher homework management on student autonomous motivation for homework (directly) and student homework engagement (indirectly) was important in terms of quantity and quality. Even if things seem to be going well, they can always improve. As with parents, it is also necessary to design evidence-based interventions that facilitate the role of the teacher in the design and monitoring of homework (Pianta et al., 2012; Rosário et al., 2015).

Given that the real involvement (of parents and teachers) may be different from students’ perceptions, we must train parents and teachers in effective ways of involvement that promote students’ competences and autonomy, and that facilitate student’s accurate perceptions of this. It is useless for teachers and parents to become involved in student homework to promote student competence and autonomy if students cannot perceive this behavior. This is what the study from Rosário et al. (2018) suggests, concluding that preparing good tasks (homework) is important, but it is not enough. In reality, it is the students who finally have to understand the teachers’ purposes, the interest of the tasks and, of course, how useful the tasks are for the development of their own competence and autonomy.

## Conclusion

Student engagement is a very important construct for explaining student progress (and dropout) in school and extracurricular tasks (Raftery et al., 2012; Rumberger and Rotermund, 2012). Student engagement is also important for the field of homework, its relationship with learning and performance, and it is a crucial element for connecting students, schools and families (Epstein, 2011). The model developed by Connell and Wellborn (1991) clearly explains how student engagement is determined by students’ motivational processes and the context. In this study we examined the mediating role of student autonomous motivation between context and student engagement.

Despite its limitations, our work provides interesting data, and some issues which may be of interest in the field of homework. For example, given the strong relationship between student autonomous motivation for homework and student homework engagement, are they different constructs or are they part of the same construct? Our data suggest that they are different constructs but there is little difference in the fit of both models. More research on this matter would be welcome. More research is also needed in order to clarify the differences between boys and girls and between middle and junior high school students regarding their perceptions of their parents’ involvement in homework. Likewise, we think that the positive, significant relationship between students’ perception of the involvement of parents and teachers in homework is very good news. This means that despite the difficulty of the connection between family and school, at least in the field of homework, there is a strong relationship: the better the perception of teacher homework management, the better the perception of parents’ content-oriented support. Although, as we see, there is already a certain connection between school and family, schools do need to think creatively about how to involve families more in educational work with their children (Raftery et al., 2012). A good example may be the approach developed by the National Network of Partnerships Schools (NNPS).

## Ethics Statement

The data of the target variables were collected during regular school hours, after obtaining the written informed consent of the school directors and the students’ parents. The questionnaires were administered in a single session by specialized staff that collaborated in the investigation. Participants completed all the questionnaires individually and without time limit. The procedures followed in the study were in accordance with the ethical standards of the Helsinki Declaration, and were approved by the Research and Teaching Ethics Committee of the University of A Coruña.

## Author Contributions

JN, BR, NS, and AV contributed conception and design of the study. IP and MR organized the database. JN and AV performed the statistical analysis. JN, BR, and NS wrote the first draft of the manuscript. IP, MR, and AV wrote sections of the manuscript. All authors contributed to manuscript revision, read and approved the submitted version.

## Conflict of Interest Statement

The authors declare that the research was conducted in the absence of any commercial or financial relationships that could be construed as a potential conflict of interest.

## Appendix: Items Used as Observed Variables

### Perceived Teacher Homework Involvement (Homework Management)

• The teachers ensure that I understand the assigned homework.
• The teachers adapt the difficulty of the homework to each of us.
• In class, we correct the homework to see where we have made mistakes.

### Perceived Parental Homework Involvement (Content-Oriented Support)

• My parents ask me if I need help with my homework.
• Generally, one of my parents helps me with my homework if I need it.
• When I have doubts about the homework, my parents’ explanations are very useful.

### Student Homework Autonomous Motivation

• I do homework with interest because it helps me to better master what the teacher explains in class every day.
• Homework is a great opportunity to check to what extent I have mastered knowledge of the subjects.
• I like doing homework because I almost always end up with a good feeling of competence and I feel proud of myself.

### Student Homework Engagement (SRL Strategies Management)

• When I’m doing homework, I think about how I’m doing it to confirm whether I am applying what the teacher taught us in class, and if not, to see how I can do better.
• Before I do the homework, I tend to think whether I am clear about what was taught in class and, if not, I review the lesson before beginning.
• Before I do my homework, I think of different ways to do it, whether I understand what I am doing, and whether I know how to apply it to other similar but unresolved classroom tasks (other problems, another text commentary, etc.).
